# Associations between self-perception of weight, food choice intentions, and consumer response to calorie information: a retrospective investigation of public health center clients in Los Angeles County before the implementation of menu-labeling regulation

**DOI:** 10.1186/s12889-016-2714-9

**Published:** 2016-01-22

**Authors:** Roch A. Nianogo, Tony Kuo, Lisa V. Smith, Onyebuchi A. Arah

**Affiliations:** 1 Department of Epidemiology, UCLA Fielding School of Public Health, 650 Charles E. Young Drive South, Los Angeles, 90095-1772 CA USA; 2 UCLA Center for Health Policy Research, Los Angeles, CA USA; 3 Division of Chronic Disease and Injury Prevention, Los Angeles County Department of Public Health, Los Angeles, CA USA; 4 Department of Family Medicine, David Geffen School of Medicine at UCLA, Los Angeles, CA USA; 5 Office of Health Assessment and Epidemiology, Los Angeles County Department of Public Health, Los Angeles, CA USA; 6 California Center for Population Research, UCLA, Los Angeles, CA USA

**Keywords:** Calorie information, Menu labeling, Obesity, Food choice, Self-perceived weight

## Abstract

**Background:**

Although obesity continues to rise and remains a great public health concern in the U.S., a number of important levers such as self-perception of weight and calorie postings at point-of-purchase in restaurants are still not well-characterized in the literature, especially for low-income and minority groups in Los Angeles County (LAC). To study this gap, we examined the associations of self-perception of weight (as measured by body weight discrepancy) with food choice intentions and consumer response to calorie information among low-income adults residing in LAC during the pre-menu labeling regulation era.

**Methods:**

Descriptive and multivariable logistic regression analyses were performed to examine the aforementioned associations utilizing data from the 2007–2008 Calorie and Nutrition Information Survey (CNIS). The CNIS was a local health department study of 639 low-income adults recruited from five large, multi-purpose public health centers in LAC.

**Results:**

Survey participants who reported that their desired weight was less than their current weight (versus desired weight the same as current weight) had (i) higher odds of intending to select lower-calorie foods under the scenario that calorie information was available at point-of-purchase (aOR = 2.0; 95 % CI: 1.0–3.9); and (ii) had higher odds of reporting that it is “very important” to have these calorie postings on food items in grocery stores (aOR = 3.1; 95 % CI: 0.90–10.7) and in fast-food restaurants (aOR = 3.4; 95 % CI: 1.0–11.4).

**Conclusions:**

Self-perception of weight was found to be associated with the intention to select lower-calorie foods under the scenario that calorie information was available at point-of-purchase. Future public health efforts to support menu labeling implementation should consider these and other findings to inform consumer education and communications strategies that can be tailored to assist restaurant patrons with this forthcoming federal law.

**Electronic supplementary material:**

The online version of this article (doi:10.1186/s12889-016-2714-9) contains supplementary material, which is available to authorized users.

## Background

Obesity remains a great public health concern in the U.S., as its prevalence continues to rise among Americans [1, 2]. In 2011–2012, almost two-thirds of Americans were overweight and obese [2], with the burden disproportionately affecting low-income and minority populations including Hispanics and African-Americans [2].

During the past decade, numerous policy, systems and environmental (PSE) change strategies have been implemented by public health authorities to combat this obesity epidemic [3–5]. These strategies have included interventions that were designed to assist individuals in losing or maintaining a normal weight through modifications of the food and the built environment [3]. For example, U.S. laws requiring menu labeling have been enacted to publicly inform food selection at the point-of-purchase in retail establishments. These laws require all restaurant chains with 20 or more outlets located in a defined geographic region to post calorie information on food items in their menus and on their menu boards. This regulatory policy first became law in several local jurisdictions across the U.S., followed later by state regulation in California under Senate Bill 1420 [6]. Menu labeling became federal law in 2010 under the Patient Protection and Affordable Care Act (ACA) [7]. Despite being well promoted initially in jurisdictions like New York City and in California, convincing data about the impact of menu labeling on consumer choice have been mixed [8, 9]. Public health authorities have conjectured that cognitive and other psychosocial factors may play critical roles in influencing food choice at point-of-purchase.

One such factor, self-perception of weight, represents a potential individual-level lever that can be used to facilitate obesity prevention efforts. Perceived or self-identity based on weight is a construct that is often operationalized as the degree of discrepancy between ideal and actual body weight [10, 11]. Some studies have shown that self-perception of weight (i.e., individuals who self-identify as normal weight, overweight or obese) represents an important measure and indicator for predicting success of behavioral interventions designed to reduce obesity in the community [12, 13]. Although recent research has shown associations between misperception of weight and weight-related behaviors [14, 15], similar investigations of these associations in low-income and minority populations in the U.S. have been more limited, especially for time periods prior to menu labeling regulation. Studying these associations may be challenging in the present because of the broad reach and the level of awareness of menu labeling in the population today.

To address this gap in the literature, a retrospective investigation (i.e., before menu labeling became a commonplace) may be better suited as a study design. An advantage of such an analysis is that it can add value and context to the evidence base, offering a historical reference and motivation for subsequent comparative studies on menu labeling. The present study sought to accomplish this by examining the associations of self-perception of weight (as measured by body weight discrepancy) with food choice intentions and consumer response to calorie information. The study utilized survey data from a sample of low-income adults residing in Los Angeles County during the pre-menu labeling regulation era.

## Methods

### Study design

Data from the Calorie and Nutrition Information Survey (CNIS) was used to perform the study analysis. The CNIS is a cross-sectional study that was conducted by the Los Angeles County Department of Public Health between 2007 and 2008 using a systematic serial sampling protocol [16]. Details of the study sample and data collection have been published elsewhere [16].

The 2008 survey remains one of few studies in Los Angeles County that examined perceptions, attitudes and consumer response to food labeling in a low-income population prior to the implementation of the state and federal menu labeling laws.

### Participants and recruitment

A total of 639 patients aged 15 to 75 years were recruited from public health centers that routinely provide tuberculosis, sexually transmitted diseases, and immunization services. In addition to age and location, prospective participants were included if they spoke English or Spanish—the third inclusion criterion. For adolescents less than 18 years of age but older than 12, parental consent was not required to receive care at the health centers. In total, there were 12 adolescents enrolled in the study; the analysis including and excluding these participants did not significantly alter the results or data interpretation; so we included them in the final analysis. Informed consent was obtained from all participants prior to enrollment and completion of the self-administered, 13-question, multi-item questionnaire. The survey response rate was estimated to be 88 % (639 out of 726 center clients approached). All study protocols, instruments and related materials were approved by the Los Angeles County Department of Public Health Institutional Review Board (IRB) prior to fieldwork. The study was considered exempt by the UCLA IRB.

### Measures

The present analysis examined the associations of self-perception of weight as measured by body weight discrepancy (BWD) with food choice intentions and consumer response to calorie information. Response to calorie information was assessed by two sets of variables measuring perception of calorie information posting and use of calorie information posting.

### Exposure variable: Body weight discrepancy

Participants were asked how much they weigh and how much they would like to weigh. The self-reported current weight (also referred to as self-reported actual weight) and the self-reported desired weight (also referred to as self-reported ideal weight) were used to create the self-perception of weight construct. This construct was measured using the body weight discrepancy (BWD) defined as the percent difference between self-reported desired and self-reported current weight. The body weight discrepancy is calculated by subtracting self-reported current weight from self-reported desired weight and dividing the difference by self-reported current weight (in pounds). This calculation of weight discrepancy was akin to what was done by Kuk et al. (2009) [17]. An absolute value of this relative difference greater or equal to 5 % was considered a meaningful difference. The 5 % cut-off was used in Jackson et al’s study (2013) to assess the desire to weigh less using the ideal-actual weight discrepancy [18]. Three categories were created: (i) desired weight less than current weight (i.e., relative difference ≤ −0.05); (ii) desired weight greater than current weight (i.e., relative difference ≥ 0.05); and (iii) desired weight same as current weight (i.e., −0.05 < relative difference < 0.05). This method was preferred for its simplicity and utility and for being rather conservative compared to the crude difference between self-reported ideal and actual weight. Other BWD formulas based on different cut-offs (i.e., 0 and 2 %) were explored in subsequent sensitivity analyses, which are included in the Additional file 1.

### Food choice intentions

Participants reported how they would use available calorie information in deciding what to order if the number of calories for foods and drinks at restaurants were listed next to each item on the menu. This food choice intention variable (outcome 1) had three response categories (i.e., would order foods and drinks with more calories; would order foods and drinks with less calories; wouldn’t change what foods and drinks I order) but was dichotomized to avoid sparse data issues as ‘*would order foods and drinks with fewer calories*’ versus ‘*would order food and drinks with more or similar calories*’.

### Perception of calorie information postings

Three variables were used to assess consumer perception of calorie information posting. Participants reported *‘yes’* or *‘no’* when asked whether they thought that fast-food and chain restaurants should have to place calorie information next to foods and drinks they serve on their menus and menu boards (outcome 2). In addition, participants reported (a) the degree to which it is important to have calorie information listed on packages and cans of food and drinks sold in grocery stores (outcome 3) and (b) the degree to which it is important to have calorie information listed on the menus or menu boards next to the foods and drinks sold in individual fast-food or chain restaurants (outcome 4). These Likert scales ranged from: ‘*very important’, ‘important’,* ‘s*omewhat important’, to ‘not important at all’*.

### Use of calorie information postings

Participants reported the frequency with which they looked at calorie information on packages and cans of foods and drinks sold in grocery stores when buying something for the first time (outcome 5). This Likert scale ranged from ‘*always’, ‘most of the time’,* ‘sometimes’*, ‘rarely’ to ‘never’*.

### Adjustment variables

To obtain adjusted odds ratios, we simultaneously included measured potential confounders of the associations under consideration in the multivariable regression modeling. These adjustment variables included age (continuous), educational attainment (categorical), sex (categorical), and race/ethnicity (categorical). In addition, we included in the adjusted model self-reported actual weight (in pounds) (continuous) and self-reported desired weight (in pounds) (continuous) to further control for the association between these variables and the various outcomes of interest. This was done in order to reduce confounding in the effect estimation of BWD on the various outcomes since the self-reported desired and self-reported actual weight variables were used to create the deterministic variable BWD [19]. Adjusting for height or body mass index (BMI) did not alter the results, and they showed no additional association with the outcomes; as a result, we did not include them in the final iteration of the model.

### Data analyses

In addition to descriptive statistics, a series of multivariable logistic regression analyses was performed to examine the associations of self-perception of weight as measured by BWD on various outcomes. Specifically, we performed binary logistic regressions for binary outcomes (outcomes 1, 2). We also performed multinomial logistic regressions for the Likert-scale outcomes (outcomes 3, 4, 5) as the proportional odds assumption did not hold for these dependent variables when treated as ordinal variables. Interpretation and presentation of the estimated odds ratios coming from the multinomial regressions was based on Hosmer and colleagues work on applied logistic regression [20]. We reported crude and adjusted odds ratios along with 95 % confidence intervals. All analyses were performed using SAS version 9.3 (SAS Institute, Cary, NC, USA).

## Results

### Survey participants’ characteristics and response to calorie information

**Table 1:** Baseline characteristics of participants in the calorie and nutrition information survey

	Total^a^	
Characteristic	n	%
Total	639	100
Age (mean, SD)	34.9 (11.6)	
Sex		
Female	348	54
Male	288	45
Race/ethnicity		
White	84	13
African-American	178	28
Hispanic/Latino	272	43
Other	97	15
Educational attainment		
Less than high school	134	21
High school graduate	175	21
Some college	160	25
College graduate/postgraduate	147	23
Desired versus current weight (in pounds)		
Desired weight less than current weight	319	50
*Underweight*	0	0
*Normal weight*	64	19
*Overweight and Obese*	255	75
Desired weight greater than current weight	76	12
*Underweight*	9	11
*Normal weight*	54	68
*Overweight and Obese*	13	16
Desired weight same as current weight	163	26
*Underweight*	8	5
*Normal weight*	98	58
*Overweight and Obese*	57	34
Self-reported current weight in lbs (mean, SD)	165 (38.4)	
Self-reported desired weight in lbs (mean, SD)	151 (31.8)	

*CNIS* Calorie and Nutrition Information Survey; *SD* standard deviation; ^a^Values may not sum up to 100 % due to missing and rounded numbers

**Table 2:** Food choice intentions and response to calorie information by self-perception of weight

	Total^a^	Desired weight same as current weight	Desired weight less than current weight	Desired weight greater than current weight
	n (%)	n (%)	n (%)	n (%)
Would use calorie information to order foods and drinks with				
Same or more calories	121 (19)	42 (25)	47 (14)	32 (41)
Fewer calories	393 (62)	101 (60)	261 (77)	31 (39)
Think calorie information should be posted				
Yes	504 (79)	140 (83)	298 (87)	66 (84)
No	82 (13)	27 (16)	43 (13)	12 (15)
Important to have calorie information listed on food items in grocery stores				
Very important	282 (44)	80 (48)	169 (50)	33 (42)
Important	160 (25)	44 (26)	99 (29)	17 (22)
Somewhat important	102 (16)	29 (17)	57 (17)	16 (20)
Not important at all	40 (06)	15 (09)	13 (04)	12 (15)
Important to have calorie information listed on meal menus in fast-food and chain restaurants				
Very important	270 (42)	63 (38)	174 (51)	33 (42)
Important	183 (29)	57 (34)	103 (30)	23 (29)
Somewhat important	95 (15)	33 (20)	49 (14)	13 (16)
Not important at all	37 (06)	14 (8)	13 (4)	10 (13)
Look at calorie information on food packages sold in grocery stores				
Always	80 (13)	29 (17)	45 (13)	6 (08)
Most of the time	121 (19)	35 (21)	73 (21)	13 (16)
Sometimes	187 (29)	50 (30)	114 (33)	23 (29)
Rarely	102 (16)	28 (17)	56 (16)	18 (23)
Never	95 (15)	25 (15)	52 (15)	18 (23)

^a^Values may not sum up to 100 % due to missing and rounded numbers 

Table 1 presents the demographic characteristics of the 639 survey participants. The mean age and standard deviations were 34.9 (±11.6 years), respectively. Fifty-four percent of the sample was female. The sampled population was ethnically diverse with 71 % self-identifying as minority (African-American or Hispanic). Half of the participants had at most a high school education. Fifty-eight percent of the survey participants’ desired weight was less than their current weight. Table 2 presents participants’ food choice intentions and response to calorie information by self-perception of weight. Almost two-thirds of the sample reported that they would use calorie information to order foods and drinks with fewer calories. Approximately half of the sample reported that it is very important to have calorie information listed on food items in grocery stores and on meal menus in individual fast-food and chain restaurants.

### Association between self-perception of weight and food choice intentions

**Table 3:** Association between self-perception of weight and food choice intentions (would chose to order fewer calories vs same or more calories)

	Crude Odds Ratio	Adjusted Odds Ratio
	cOR (95%CI)	aOR (95%CI)
*Desired versus current weight (in pounds)*		
Desired weight less than current weight	2.3 (1.4, 3.7)	2.0 (1.0, 3.9)
Desired weight greater than current weight	0.4 (0.2, 0.7)	0.5 (0.3, 1.1)
Desired weight same as current weight	1.0	1.0

*cOR* crude odds ratio; *aOR* adjusted odds ratio; *CI* confidence interval; *Ref*, reference

The odds of intending to use calorie information to order food and drinks with fewer calories among those whose desired weight was less than their current weight were two-fold those of participants whose desired weight was the same as their current weight (aOR: 2.0; 95 % CI:1.0, 3.9).(Table 3). 

### Association between self-perception of weight and response to calorie information posting

**Table 4:** Association between self-perception of weight and response to calorie information posting

	Crude Odds Ratio	Adjusted Odds Ratio
	cOR (95%CI)	aOR (95%CI)
Perception of calorie information posting		
Outcome 2: Think calorie information should be posted		
(Yes vs No)		
*Desired versus current weight (in pounds)*		
Desired weight less than current weight	1.2 (0.8, 1.8)	1.5 (0.8, 2.7)
Desired weight greater than current weight	1.5 (0.8, 2.8)	1.2 (0.6, 2.5)
Desired weight same as current weight	1.0	1.0
Outcome 3: Important to have calorie information listed on food items in grocery stores		
(Ref = Not important at all)		
*Desired versus current weight (in pounds)*		
Desired weight less than current weight		
*Very important*	2.4 (1.1, 5.4)	3.1 (0.9, 10.7)
*Important*	2.6 (1.1, 5.9)	3.7 (1.1, 13.3)
*Somewhat important*	2.3 (1.0, 5.4)	3.0 (0.8, 11.2)
Desired weight greater than current weight		
*Very important*	0.5 (0.2, 1.2)	0.4 (0.2, 1.2)
*Important*	0.5 (0.2, 1.2)	0.3 (0.1, 1.1)
*Somewhat important*	0.7 (0.3, 1.8)	0.5 (0.2, 1.7)
Desired weight same as current weight	1.0	1.0
Outcome 4: Important to have calorie information listed on meal menus in fast-food and chain restaurants		
(Ref = Not important at all)		
*Desired versus current weight (in pounds)*		
Desired weight less than current weight		
*Very important*	3.0 (1.3, 6.7)	3.4 (1.0, 11.4)
*Important*	1.9 (0.9, 4.4)	2.9 (0.9, 9.9)
*Somewhat important*	1.6 (0.7, 3.8)	2.2 (0.6, 8.0)
Desired weight greater than current weight		
*Very important*	0.7 (0.3, 1.8)	0.7 (0.2, 2.3)
*Important*	0.6 (0.3, 1.8)	0.4 (0.1, 1.4)
*Somewhat important*	0.6 (0.2, 1.5)	0.5 (0.1, 1.6)
Desired weight same as current weight	1.0	1.0
Use of calorie information posting		
Outcome 5: Look at calorie information on food packages sold in grocery stores		
(Ref = Never)		
*Desired versus current weight (in pounds)*		
Desired weight less than current weight		
*Always*	0.7 (0.4, 1.5)	1.2 (0.5, 3.2)
*Most of the time*	1.0 (0.5, 1.9)	1.8 (0.8, 4.3)
*Sometimes*	1.1 (0.6, 2.0)	1.5 (0.7, 3.3)
*Rarely*	1.0 (0.5, 1.9)	1.0 (0.4, 2.4)
Desired weight greater than current weight		
*Always*	0.3 (0.1, 0.8)	0.3 (0.1, 1.0)
*Most of the time*	0.5 (0.2, 1.2)	0.6 (0.2, 1.7)
*Sometimes*	0.6 (0.3, 1.4)	0.7 (0.3, 1.7)
*Rarely*	0.9 (0.4, 2.1)	0.9 (0.3, 2.3)
Desired weight same as current weight	1.0	1.0

*cOR* crude odds ratio; *aOR* adjusted odds ratio; *CI* confidence interval; *Ref* reference

Participants whose desired weight was less than their current weight had odds of 1.5 (95 % CI: 0.8, 2.7) of perceiving that fast-food and chain restaurants should have to place calorie information next to foods and drinks they serve on menus and menu boards compared to those whose desired weight was the same as their current weight (Table 4). The odds of reporting that it is ‘very important’ and ‘important’ to have calorie information on food items in grocery stores and in individual fast-food and chain restaurants relative to reporting that it is not important at all, among participants whose desired weight was less than their current weight were respectively 3.1 (95 % CI: 0.9, 10.7), 3.7 (95 % CI: 1.1, 13.3), 3.4 (95 % CI: 1.0, 11.4) and 2.9 (0.9, 9.9) times those of participants who reported the same desired weight as their current weight. The odds of reporting ‘always’ and ‘most of the time’ looking at calorie information on food packages sold in grocery stores relative to reporting never, among participants whose desired weight was less than their current weight were respectively 1.2 (95 % CI: 0.5, 3.2) and 1.8 (95 % CI: 0.8, 4.3) times those of participants who reported the same desired weight as their current weight.

## Discussion

The purpose of this study was to examine the associations of self-perception of weight (as measured by BWD) with food choice intentions and consumer response to calorie information among low-income adults residing in Los Angeles County during the pre-menu labeling regulation era. Our findings suggest that adults whose desired weight was less than their current weight had higher odds of intending to use calorie information to select lower-calorie foods if point-of-purchase calorie postings were available and higher odds of perceiving that it was important to have calorie information on food items in grocery stores and individual fast-food and chain restaurants. The study also found that self-perception of weight did not appear to be associated with the frequency of use of calorie information.

The perception of one’s own weight relative to one’s ideal or desired weight may represent a useful indicator of how a person might select lower-calorie foods in the retail setting if he/she was exposed to calorie information at point-of-purchase. A number of studies have shown that individuals who self-identify as overweight or obese (such as when desired weight is less than current weight) may be inclined to engage in weight management behaviors such as reducing the calories consumed in a given day [12, 13]. Likewise, an inadequate perception of weight, especially for those who are overweight and obese can hinder initiation to lifestyle change [21]. In light of this, our findings have important implications for the menu labeling change strategy that is being implemented nationally. First, people who perceive that their desired weight is less than their current weight [i.e., perceived susceptibility construct of the Health Belief Model (HBM) [22]] and who understand the importance of menu labeling (i.e., perceived benefit construct of the HBM), may be a step closer to using calorie information for weight-loss behaviors than those whose desired weight is more than or the same as their current weight. Second, the future success of menu labeling may be correlated with how individuals perceive or will perceive their own weight as is relates to their ideal weight. In other words, environmental strategies such as point-of-purchase calorie information, in the absence of other strategies that target psychosocial factors, food access or pricing, are arguably not standalone interventions. Rather, they are strategies that can synergistically promote healthy eating when paired with these other efforts [23]. A recent review outlined that certain combinations of community-based restaurant interventions such as point-of-purchase information may lead to healthy food selection in the presence of increased availability of healthy choices [23].

Contrary to our expectations, individuals whose desired weight was less than their current weight did not frequently look at calorie information on food packages when purchasing them the first time. This is surprising since individuals in this category have reported that it is important to have calorie information posted in the retail setting. This could presage that individuals whose desired weight is less than their current weight may actually not use calorie information to select lower-calorie information even if calorie information is made available at point-of-purchase. Reasons for why individuals may not use calorie information have been documented [24]. They include low prior knowledge of the meaning of nutrients and daily reference values, the frustration with deciphering nutritional information, and dealing with competing factors such as price, discounts and promotion [24].

These aforementioned knowledge and financial barriers may be more pronounced among low-income populations like the sample in our study and may have contributed in part to our findings [25]. To the best of our knowledge, the present study is one of the first that has looked at the associations of self-perception of weight with food choice intentions and consumer response to calorie information in a low-income population. Such research has been lacking in more homogenous and higher-income groups, suggesting that more studies are needed to understand the dynamics of self-perception of weight as it relates to menu labeling in this group. For higher-income groups, there are reasons to believe that findings will be different from lower income groups [26]. Collectively, these and other results suggest that more should be done to educate consumers about menu labeling, nutrition labels in stores, and dietary references—all of these can have an impact on food choice among the intended audiences [25].

### Strength and limitations

The strengths of the present study and its analytic approach include: i) the use of the CNIS dataset, which was collected before the implementation of the 2008 menu labeling requirement (SB 1420) and which contains information on participants who were from ethnically diverse low-income populations in Los Angeles County; ii) the availability and breadth of collected constructs—perception, attitudes and behavior—pertaining to consumer response to calorie information postings; and iii) the inclusion of a series of sensitivity analyses in the overall execution of the analyses.

That being said, the CNIS and the present analysis have a number of notable limitations. First, the CNIS data is 7 years old and findings from this study may reflect different attitudes and behaviors that may have changed if the survey was repeated today. However, the CNIS data represents a historical context of the pre-menu labeling era, that is, before menu labeling became widely accepted and adopted. Data collected during such period offers rich insights and baseline benchmarking which are critical to answering the research question(s). They provide a reference for subsequent comparative studies on the subject matter, as the federal menu labeling law rolls out over the next several years. Second, study findings may be subject to the presence of residual but minimal confounding since we did not have all of the information on participants, including their income. Third, we believe selection bias is possible but minimal or absent in this study since neither self-perception of weight nor consumer response to calorie information would be expected to directly influence participation in the study. In addition, the study response rate was relatively high (88 %) [16]. Additionally, the study population may also not be representative of the general population, but rather of the low-income and uninsured or underinsured population who frequent public health centers. Finally, as it is common in most surveys, our findings, could have been affected by measurement error due to self-reported data. Several studies have reported that individuals who are typically female or overweight and/or obese tend to underreport their weight [27, 28]. This would likely underestimate the true relationship between BWD and the various outcomes studied, suggesting that the true estimate are even higher than reported in the present study. More validation studies or studies that collect measured weights and heights should be conducted to assess the true magnitude of these estimates. It is possible also that the definition of BWD may have resulted in misclassification of exposure and affected the findings and interpretations. To check this, we conducted a wide-ranging set of sensitivity analyses with several definitions of BWD; most results and interpretations did not substantially change (see Additional file 1).

## Conclusions

Self-perception of desired weight vis-à-vis current weight (BWD) may be predictive of intention to select lower-calorie foods if calorie information were made available at point-of-purchase to public health center clients in the retail setting. Using data that was collected before menu labeling was implemented in Los Angeles County offered a reference for future comparative work on these aforementioned associations. Future public health efforts to support menu labeling implementation in Los Angeles and elsewhere in the U.S. should consider these and other research findings to inform consumer education and communications strategies that can be tailored to assist restaurant patrons use calorie information and prepare for the forthcoming roll-out of the ACA menu labeling requirements.

## Additional file

Additional file 1:
**Sensitivity analysis.** (DOCX 27 kb)

## Abbreviations

BWD: Body weight discrepancy
CNIS: Calorie and nutrition information survey
PSE: Policy, systems and environmental change strategies
ACA: Patient Protection and Affordable Care Act of 2010
