# Supplementary material for: Associations between self-perception of weight, food choice intentions, and consumer response to calorie information: a retrospective investigation of public health center clients in Los Angeles County before the implementation of menu-labeling regulation
Source: BMC Public Health. 2016 Jan 22;16:60. doi: 10.1186/s12889-016-2714-9 (PMC4722675; doi:10.1186/s12889-016-2714-9)
Supplement: Additional file 1: — Sensitivity analysis. (DOCX 27 kb) [file 12889_2016_2714_MOESM1_ESM.docx]

**Sensitivity analysis**

To examine the potential influence of BWD on the outcome variables, additional analyses were carried out using different definitions for the discrepancy (BWD) variable. The definition used in the main analysis is based on the relative difference between desired and current weight in pounds (Definition 1). Here, we present subsequent analyses using other definitions of the BWD based different cut-offs (i.e., 0% and 2%). In particular, under Definition 2, BWD or ‘desired versus current weight’ was defined as the difference between self-reported desired (ideal) weight and self-reported current (actual) weight, all measured in pounds. Three categories were created using a zero difference cutoff: (i) desired weight less than current weight (i.e., difference < 0); (ii) desired weight greater than current weight (i.e., difference > 0) and (iii) desired weight same as current weight (i.e., difference = 0). The third definition, similar to the first definition used in the main manuscript used a cut-off of 2% instead of 5% for the categorization of BWD. Three categories were created: (i) desired weight less than current weight (i.e., relative difference ≤ - 0.02); (ii) desired weight greater than current weight (i.e., relative difference ≥ 0.02); and (iii) desired weight same as current weight (i.e., -0.02 < relative difference < 0.02)

**Additional file 1 (definition 2)**

**Table S1.** Association between self-perception of weight and food choice intention in a public health center population, Los Angeles County, 2007-2008 (n = 639)

|  | Crude Odds Ratio  (cOR: 95%CI) | Adjusted Odds Ratio  (aOR:95%CI) |
| --- | --- | --- |
| Outcome 1: Would use calorie information to order foods and drinks with (Fewer calories vs same or more calories) |  |  |
| Desired versus current weight (in pounds) |  |  |
| Desired weight less than current weight | 1.7 ( 0.9,3.1) | 1.2 ( 0.6,2.5) |
| Desired weight greater than current weight | 0.4 ( 0.2,0.8) | 0.6 ( 0.3,1.3) |
| Desired weight same as current weight | 1 | 1 |

cOR = crude odds ratio ; aOR = adjusted odds ratio; CI: confidence interval; Ref = reference

**Table S2**. Association between self-perception of weight and response to calorie information posting in a public health center population, Los Angeles County, 2007-2008 (n = 639)

|  | Crude Odds Ratio  (cOR: 95%CI) | Adjusted Odds Ratio  (aOR:95%CI) |
| --- | --- | --- |
| Outcome 2: Calorie information should be posted  (Yes vs No) |  |  |
| Desired versus current weight (in pounds) |  |  |
| Desired weight less than current weight | 1.5 ( 0.9,2.6) | 2 ( 1.0, 3.8) |
| Desired weight greater than current weight | 1.6 ( 0.8,3.1) | 1.3 ( 0.6,2.6) |
| Desired weight same as current weight | 1 | 1 |
|  |  |  |
| Outcome 3: Important to have calorie information listed on food items in grocery stores (Ref = Not important at all) |  |  |
| Desired versus current weight (in pounds) |  |  |
| Desired weight less than current weight |  |  |
| Very important | 2.6 ( 1.1,6.2) | 2.6 ( 0.8,8.1) |
| Important | 3.2 ( 1.2,8.1) | 3.5 ( 1.1,11.8) |
| Somewhat important | 1.9 ( 0.7,5) | 1.6 ( 0.5,5.8) |
| Desired weight greater than current weight |  |  |
| Very important | 0.9 ( 0.3,2.2) | 1.0 ( 0.3,2.9) |
| Important | 1.1 ( 0.4,3.0) | 1.2 ( 0.4,3.9) |
| Somewhat important | 1.1 ( 0.4,3.1) | 1.1 ( 0.3,3.8) |
| Desired weight same as current weight | 1 | 1 |
|  |  |  |
| Outcome 4: Important to have calorie information listed on meal menus in fast food and restaurant (Ref = Not important at all) |  |  |
| Desired versus current weight (in pounds) |  |  |
| Desired weight less than current weight |  |  |
| Very important | 1.9 ( 0.7,5.2) | 1.5 ( 0.5,4.8) |
| Important | 1.3 ( 0.5,3.4) | 1.3 ( 0.4,4.2) |
| Somewhat important | 1.1 ( 0.4,3.3) | 1 ( 0.3,3.4) |
| Desired weight greater than current weight | 1 | 1 |
| Very important | 0.8 ( 0.3,2.5) | 1.4 ( 0.4,4.6) |
| Important | 0.6 ( 0.2,1.9) | 0.8 ( 0.2,2.7) |
| Somewhat important | 0.9 ( 0.3,2.9) | 1.2 ( 0.3,4.6) |
| Desired weight same as current weight | 1 | 1 |
|  |  |  |
| Outcome 5: Look at calorie information on food packages sold in grocery stores (Ref = Never) |  |  |
| Desired versus current weight (in pounds) |  |  |
| Desired weight less than current weight |  |  |
| Always | 0.7 ( 0.3,1.4) | 1.2 ( 0.4,3.1) |
| Most of the time | 1.5 ( 0.7,3.5) | 2.2 ( 0.8,5.9) |
| Sometimes | 1.8 ( 0.8,3.8) | 2.4 ( 1,5.8) |
| Rarely | 0.9 ( 0.4,2) | 0.8 ( 0.3,2.2) |
| Desired weight greater than current weight | 1 | 1 |
| Always | 0.3 ( 0.1,0.8) | 0.3 ( 0.1,1.1) |
| Most of the time | 1.2 ( 0.5,3) | 1.7 ( 0.6,4.8) |
| Sometimes | 1.2 ( 0.5,3) | 1.5 ( 0.6,3.8) |
| Rarely | 1 ( 0.4,2.4) | 1 ( 0.4,2.6) |
| Desired weight same as current weight | 1 | 1 |

cOR = crude odds ratio ; aOR = adjusted odds ratio; CI: confidence interval; Ref = reference

**Appendix 1 (definition 3)**

**eTable 3.** Association between self-perception of weight and food choice intention in a public health center population, Los Angeles County, 2007-2008 (n = 639)

|  | Crude Odds Ratio  (cOR: 95%CI) | Adjusted Odds Ratio  (aOR:95%CI) |
| --- | --- | --- |
| Outcome 1: Would use calorie information to order foods and drinks with (Fewer calories vs same or more calories) |  |  |
| Desired versus current weight (in pounds) |  |  |
| Desired weight less than current weight | 2.3 ( 1.3, 4) | 1.7 ( 0.8, 3.3) |
| Desired weight greater than current weight | 0.6 ( 0.3, 1.1) | 0.7 ( 0.4, 1.5) |
| Desired weight same as current weight | 1 | 1 |

cOR = crude odds ratio ; aOR = adjusted odds ratio; CI: confidence interval; Ref = reference

**eTable 4.** Association between self-perception of weight and response to calorie information posting in a public health center population, Los Angeles County, 2007-2008 (n = 639)

|  | Crude Odds Ratio  (cOR: 95%CI) | Adjusted Odds Ratio  (aOR:95%CI) |
| --- | --- | --- |
| Outcome 2: Calorie information should be posted |  |  |
| (Yes vs No) |  |  |
| Desired versus current weight (in pounds) |  |  |
| Desired weight less than current weight | 1.5 ( 0.8, 2.8) | 1.4 ( 0.6, 2.9) |
| Desired weight greater than current weight | 1.3 ( 0.6, 2.7) | 1.9 ( 0.8, 4.5) |
| Desired weight same as current weight | 1 | 1 |
|  |  |  |
| Outcome 3: Important to have calorie information listed on food items in grocery stores (Ref = Not important at all) |  |  |
| Desired versus current weight (in pounds) |  |  |
| Desired weight less than current weight |  |  |
| Very important | 3 ( 1.3, 7) | 3.8 ( 1.2, 12.1) |
| Important | 3.5 ( 1.4, 8.6) | 5.1 ( 1.5, 17.4) |
| Somewhat important | 2.5 ( 1, 6.3) | 2.8 ( 0.8, 10) |
| Desired weight greater than current weight |  |  |
| Very important | 0.8 ( 0.3, 2.1) | 0.8 ( 0.3, 2.4) |
| Important | 1 ( 0.4, 2.6) | 0.9 ( 0.3, 2.9) |
| Somewhat important | 1.2 ( 0.4, 3.3) | 1.1 ( 0.4, 3.6) |
| Desired weight same as current weight | 1 | 1 |
| Outcome 4: Important to have calorie information listed on meal menus in fast food and restaurant (Ref = Not important at all) |  |  |
| Desired versus current weight (in pounds) |  |  |
| Desired weight less than current weight |  |  |
| Very important | 2.4 ( 1, 6) | 2 ( 0.6, 6.5) |
| Important | 1.6 ( 0.6, 4) | 1.9 ( 0.6, 6) |
| Somewhat important | 1.5 ( 0.5, 4) | 1.5 ( 0.4, 5.3) |
| Desired weight greater than current weight |  |  |
| Very important | 0.9 ( 0.3, 2.5) | 1.2 ( 0.4, 3.8) |
| Important | 0.7 ( 0.2, 1.9) | 0.8 ( 0.2, 2.5) |
| Somewhat important | 0.9 ( 0.3, 2.9) | 1.2 ( 0.3, 4.2) |
| Desired weight same as current weight | 1 | 1 |
|  |  |  |
| Outcome 5: Look at calorie information on food packages sold in grocery stores (Ref = Never) |  |  |
| Desired versus current weight (in pounds) |  |  |
| Desired weight less than current weight |  |  |
| Always | 0.7 ( 0.3, 1.4) | 1.1 ( 0.4, 2.9) |
| Most of the time | 1.3 ( 0.6, 2.7) | 1.9 ( 0.8, 4.8) |
| Sometimes | 1.8 ( 0.9, 3.7) | 2.6 ( 1.1, 6.2) |
| Rarely | 1.1 ( 0.5, 2.4) | 1 ( 0.4, 2.7) |
| Desired weight greater than current weight |  |  |
| Always | 0.2 ( 0.1, 0.7) | 0.3 ( 0.1, 0.9) |
| Most of the time | 0.8 ( 0.3, 2.1) | 1.1 ( 0.4, 3) |
| Sometimes | 1.1 ( 0.5, 2.6) | 1.2 ( 0.5, 3) |
| Rarely | 1.1 ( 0.4, 2.7) | 1.1 ( 0.4, 2.8) |
| Desired weight same as current weight | 1 | 1 |

cOR = crude odds ratio ; aOR = adjusted odds ratio; CI: confidence interval; Ref = reference
